# Supplementary material for: Retinal Dystrophy Associated with Homozygous Variants in NRL
Source: Genes (Basel). 2024 Dec 12;15(12):1594. doi: 10.3390/genes15121594 (PMC11675615; doi:10.3390/genes15121594)
Supplement: Supplementary file 1 [file genes-15-01594-s001.zip › Supplementary_Figures_S1-S4.pdf]

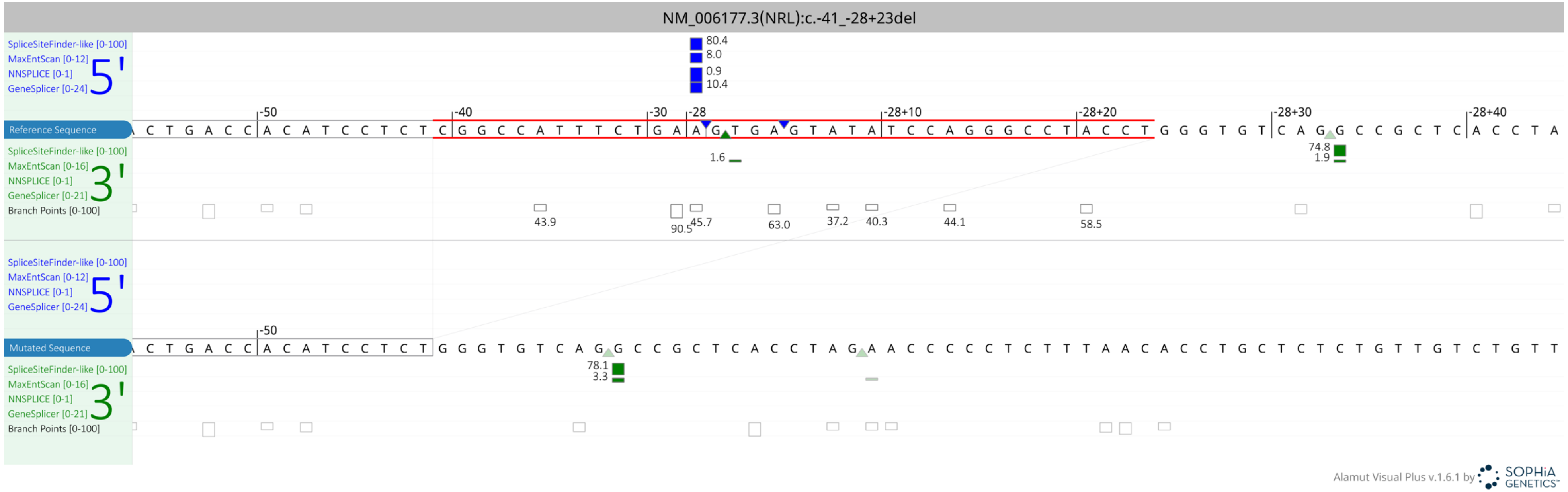

Figure S1. Visual representation of splicing prediction algorithms from the candidate variant in *NRL* (NM_006177.3:c.-41_-28+23del). The figure was created from a screenshot of the Alamut Visual Plus software. The panel shows the genomic region surrounding the variant (reference sequence above the variant sequence) with the respective splice site predictions computed by the algorithms included in Alamut Visual Plus (SpliceSiteFinder-like, MaxEntScan, NNSPLICE, and GeneSplicer). Predicted acceptor and donor splice sites are represented by green and blue shapes, respectively. The red lines highlight the deleted sequence.

A)

B)

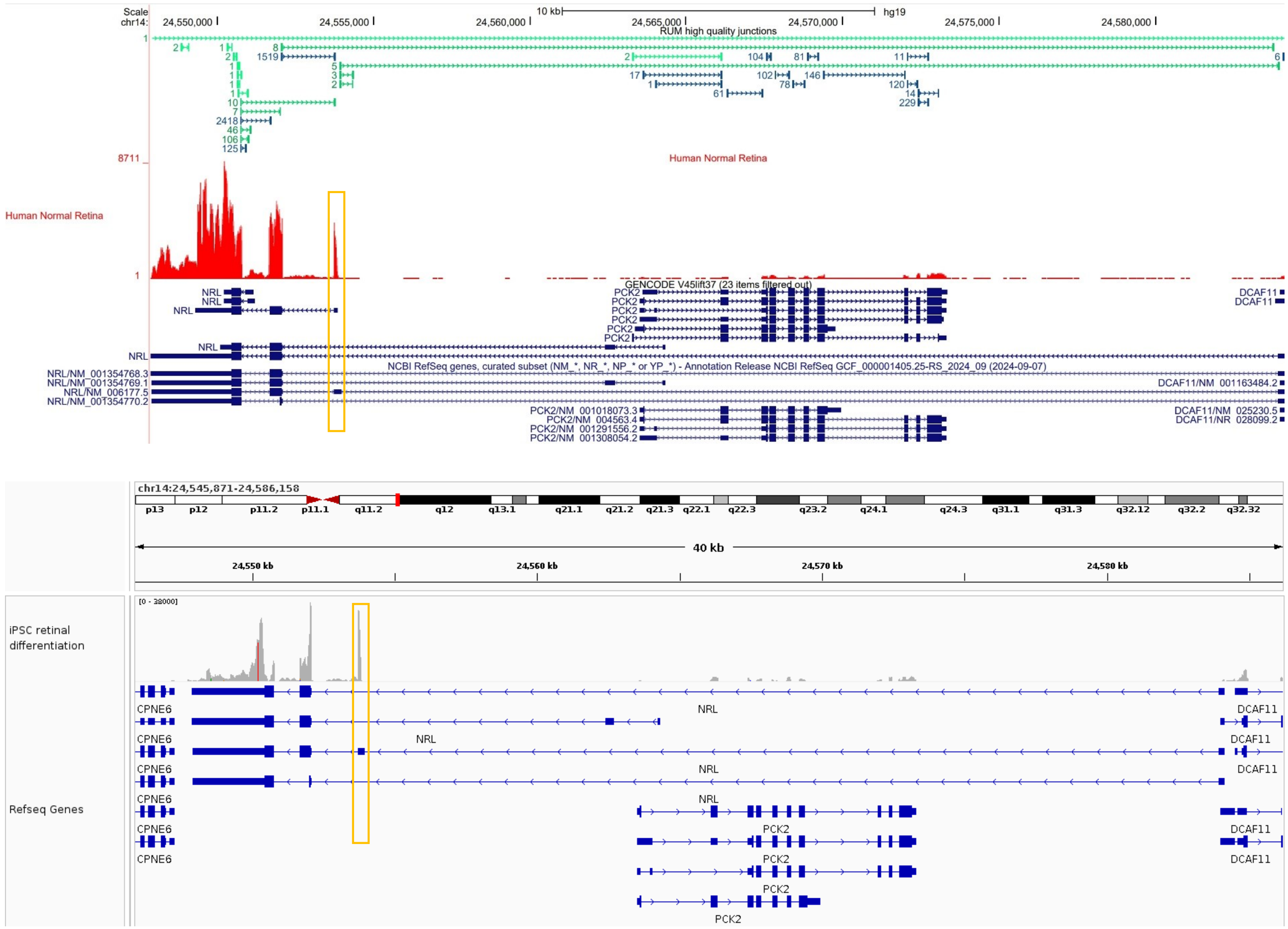

Scale
chr14:
10 kb
hg19
24,550,000
24,555,000
24,560,000
24,565,000
24,570,000
24,575,000
24,580,000
RUM high quality junctions
8711
Human Normal Retina
Human Normal Retina
GENCODE V45lift37 (23 items filtered out)
NRL
PCK2
DCAF11
NCBI RefSeq genes, curated subset (NM_*, NR_*, NP_* or YP_*) - Annotation Release NCBI RefSeq GCF_000001405.25-RS_2024_09 (2024-09-07)
NRL/NM_001354768.3
NRL/NM_001354769.1
NRL/NM_006177.5
NRL/NM_001354770.2
DCAF11/NM_001163484.2
PCK2/NM_001018073.3
PCK2/NM_004563.4
PCK2/NM_001291556.2
PCK2/NM_001308054.2
DCAF11/NM_025230.5
DCAF11/NR_028099.2
chr14:24,545,871-24,586,158
p13
p12
p11.2
p11.1
q11.2
q12
q13.1
q21.1
q21.2
q21.3
q22.1
q22.3
q23.2
q24.1
q24.3
q31.1
q31.3
q32.12
q32.2
q32.32
40 kb
24,550 kb
24,560 kb
24,570 kb
24,580 kb
[0 - 38000]
iPSC retinal differentiation
Refseq Genes
CPNE6
NRL
DCAF11
PCK2

Figure S2. Visual representation of RNA-seq data coverage for the genomic region including the *NRL* gene. These coverage plots suggest that transcript NM_006177.3 (or possibly NM_006177.5) is the main transcript in the retina. A) Coverage of RNA-seq data generated from healthy donor retinae from another group (https://oculargenomics.meei.harvard.edu/retinal-transcriptome/) as shown on the UCSC Genome Browser. The orange frame highlights the region of NM_006177.3 exon 1 or NM_006177.5 exon 2. B) Coverage of RNA-seq data generated from differentiated human retinal organoids. The orange frame highlights the region of NM_006177.3 exon 1 or NM_006177.5 exon 2.

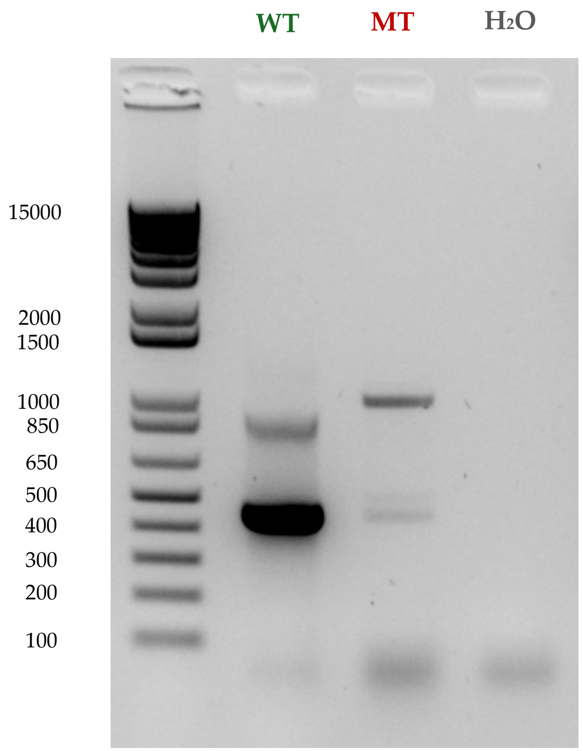

Figure S3. Gel electrophoresis from the reference and variant minigenes for the *NRL* variant NM_006177.3:c.-41_-28+23del. Abbreviations: WT, wildtype (reference) minigene; MT, mutant (variant) minigene; H_2O, water control.

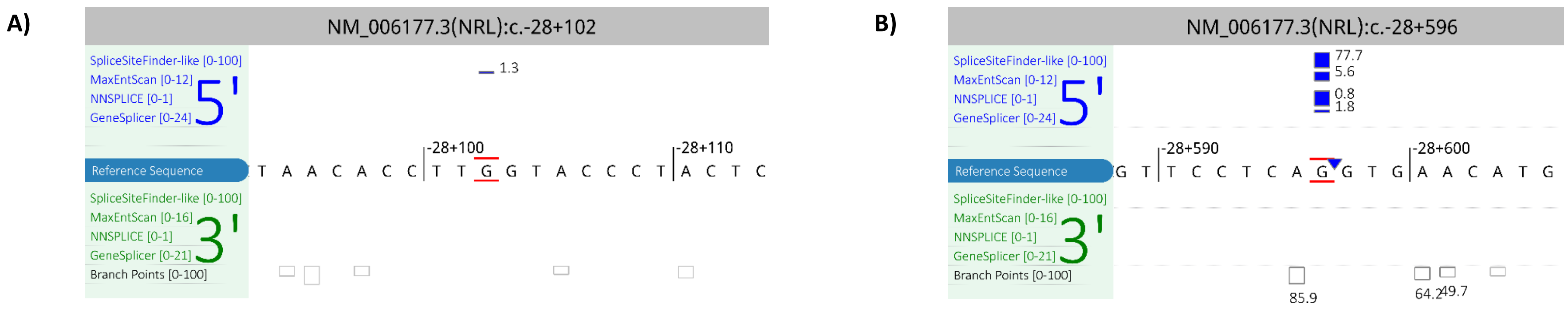

Figure S4. Visual representation of splicing prediction algorithms from the alternative cryptic donor splice sites utilized in aberrant transcripts T2 and T3. The figure was created from a screenshot of the Alamut Visual Plus software. The panel shows the genomic region surrounding the respective cryptic donor splice site and the predictions computed by the algorithms included in Alamut Visual Plus (SpliceSiteFinder-like, MaxEntScan, NNSPLICE, and GeneSplicer). Predicted donor splice sites are represented by blue shapes. A) Predictions for cryptic donor splice site at position c.-28+102 suggest it to be weak. B) Predictions for cryptic donor splice site at position c.-28+596 suggest it to be strong.
